# *Fusobacterium nucleatum* induces a tumor microenvironment with diminished adaptive immunity against colorectal cancers

**DOI:** 10.3389/fcimb.2023.1101291

**Published:** 2023-03-07

**Authors:** Han Sang Kim, Chang Gon Kim, Won Kyu Kim, Kyung-A Kim, Jinseon Yoo, Byung Soh Min, Soonmyung Paik, Sang Joon Shin, Hyukmin Lee, Kyungwon Lee, Hoguen Kim, Eui-Cheol Shin, Tae-Min Kim, Joong Bae Ahn

**Affiliations:** ^1^ Yonsei Cancer Center, Division of Medical Oncology, Department of Internal Medicine, Yonsei University College of Medicine, Seoul, Republic of Korea; ^2^ Graduate School of Medical Science, Brain Korea 21 FOUR Project for Medical Science, Yonsei University College of Medicine, Seoul, Republic of Korea; ^3^ Natural Products Research Center, Korea Institute of Science and Technology, Gangneung, Republic of Korea; ^4^ Department of Medical Informatics, College of Medicine, The Catholic University of Korea, Seoul, Republic of Korea; ^5^ Department of Surgery, Yonsei Cancer Center, Yonsei University College of Medicine, Seoul, Republic of Korea; ^6^ Department of Laboratory Medicine, Yonsei University College of Medicine, Seoul, Republic of Korea; ^7^ Department of Pathology, Severance Hospital, Yonsei University College of Medicine, Seoul, Republic of Korea; ^8^ Graduate School of Medical Science and Engineering, Korea Advanced Institute of Science and Technology, Daejeon, Republic of Korea

**Keywords:** anti-tumor immune response, T cells, gut microbiota, Fusobacterium nucleatum, colorectal cancer

## Abstract

**Background & Aims:**

*Fusobacterium nucleatum* (FN) plays a pivotal role in the development and progression of colorectal cancer by modulating antitumor immune responses. However, the impact of FN on immune regulation in the tumor microenvironment has not been fully elucidated.

**Methods:**

The abundance of FN was measured in 99 stage III CRC tumor tissues using quantitative polymerase chain reaction. Gene expression profiles were assessed and annotated using consensus molecular subtypes (CMS), Gene Ontology (GO) analysis, and deconvolution of individual immune cell types in the context of FN abundance. Immune profiling for tumor infiltrating T cells isolated from human tumor tissues was analyzed using flow cytometry. *Ex vivo* tumor-infiltrating T cells were stimulated in the presence or absence of FN to determine the direct effects of FN on immune cell phenotypes.

**Results:**

Gene expression profiles, CMS composition, abundance of immune cell subtypes, and survival outcomes differed depending on FN infection. We found that FN infection was associated with poorer disease-free survival and overall survival in stage III CRC patients. FN infection was associated with T cell depletion and enrichment of exhausted CD8^+^ and FoxP3^+^ regulatory T cells in the tumor microenvironment. The presence of FN in tumors was correlated with a suppressive tumor microenvironment in a T cell-dependent manner.

**Conclusion:**

FN enhanced the suppressive immune microenvironment with high depletion of CD8^+^ T cells and enrichment of FoxP3^+^ regulatory T cells in human colorectal cancer cases. Our findings suggest a potential association for FN in adaptive immunity, with biological and prognostic implications.

## 1 Introduction

Colorectal cancer (CRC) is the third most common cancer and second leading cause of cancer-related deaths worldwide (Sung et al., 2021). Multimodal treatments, including surgical resection, radiation therapy, chemotherapy, and, recently, immunotherapy, have led to major advances in the treatment of CRC. However, the survival outcome of patients with advanced CRC remains poor, with a 5-year survival rate of 10–20% (Siegel et al., 2020). Various factors are involved in determining the prognosis and treatment outcome of individual patients with CRC, including tumor-intrinsic genomic and molecular signatures (Cancer Genome Atlas, 2012), transcriptional profiles (Guinney et al., 2015), lifestyle or comorbidities (Van Blarigan and Meyerhardt, 2015), and immune environment and landscape (Bruni et al., 2020).

Recent estimates of the number of bacterial cells in our bodies to human cells have lowered the ratio from 10:1 (Tremaroli and Bäckhed, 2012) to 1.3:1 (Sender et al., 2016). The human gut microbiota plays an important role in homeostasis, immunity, and intestinal tumorigenesis (Hooper et al., 2012; Tremaroli and Bäckhed, 2012; Brennan and Garrett, 2019). *Fusobacterium nucleatum* (FN) is a Gram-negative anaerobic bacterium that exists in the human gastrointestinal tract, spanning the oral cavity to the anus (Rubinstein et al., 2013). FN has been identified as a potential cancer driver involved in CRC (Castellarin et al., 2012; Kostic et al., 2012; Mima et al., 2016). In The Cancer Genome Atlas (TCGA) cohort, compared to those having lower amounts of FN, poorer overall survival was observed in patients with CRC having a high amount of FN (Bullman et al., 2017). We have reported previously that FN enrichment has significantly poorer prognosis in patients with right-sided metastatic colon cancer (Lee et al., 2021). High presence of FN in CRC tumor tissue is found to be inversely correlated with T cell density (Gur et al., 2015; Saito et al., 2016; Borowsky et al., 2021). Previous studies have proven that FN affects antitumor activity of immune cells *via* activating immune checkpoints such as CEA-related cell adhesion molecule (CEACAM) (Galaski et al., 2021), T cell immunoglobulin and ITIM domain (TIGIT) (Gur et al., 2015), and programmed death ligand 1 (PD-L1) (Gao et al., 2021). Despite these findings, the relationship between FN and host immune surveillance in the colorectal tumor microenvironment remains unclear.

Herein, we investigated the crosstalk between the host adaptive immune system and FN by analyzing the gene expression profiles, immune phenotyping, and interrogation of patient prognosis. Using this approach, we reassessed FN as a major player in CRC pathogenesis and determined treatment response.

## 2 Materials and methods

### 2.1 Study design and participants

The study population comprised 99 treatment-naïve patients with CRC who underwent surgical resection between January 2006 and December 2012 (**Table 1**). All authors followed good clinical practice, and the study was conducted according to the principles of the Declaration of Helsinki. The study protocol was approved by the institutional review board of Severance Hospital (IRB no: 4-2018-0291).

**Table 1:** Patient characteristics (*n*=99).

Characteristics		Number of patients (%)
Median age and range (years)		61 (33–78)
Sex		
Male		49 (49.5%)
Female		50 (50.5%)
Stage		
3		99 (100.0%)
Location		
Right side		35 (35.4%)
Left side		64 (64.6%)
Histology		
Adenocarcinoma		93 (93.9%)
Mucinous carcinoma		4 (4.0%)
Medullary carcinoma		2 (2.0%)
Differentiation		
Well differentiated		5 (5.1%)
Moderately differentiated		88 (88.9%)
Poorly differentiated		6 (6.1%)
Lymphovascular invasion		
Absent		48 (48.5%)
Present		51 (51.5%)
Perineural invasion		
Absent		86 (86.9%)
Present		13 (13.1%)
*KRAS* mutation		
Wild-type		59 (59.6%)
Mutant-type		40 (40.4%)
*BRAF* mutation		
Wild-type		98 (99.0%)
Mutant-type		1 (1.0%)
Microsatellite instability		
MSS/MSI-L		91 (91.9%)
MSI-H		8 (8.1%)
Preoperative CEA		
≥5 ng/mL		28 (28.3%)
<5 ng/mL		71 (71.7%)

CEA, carcinoembryonic antigen.

### 2.2 Bacterial strains

The bacterial strain *F. nucleatum* (ATCC 25586) was obtained from the American Type Culture Collection (ATCC). The strain was cultured on Brucella agar plates with 5% sheep blood and incubated in a Coy chamber (Coy Laboratory Product, USA) for 3 days at 37°C under anaerobic conditions (atmosphere: 20% CO_2_, 5% H_2_, and 75% N_2_). A single colony was inoculated into a Gifu anaerobic medium (GAM) broth (KisanBio, Korea) and incubated at 37°C for 18 hr. Bacterial cells were centrifuged at 2,000 х *g* for 5 min at 4°C and washed twice with PBS. For calculating colony-forming units per millimeter (CFU/mL) of a fluid, the pellet was serially diluted 10-fold in fresh GAM broth and spread onto the Brucella agar plate. The mean colony counts were 1.0 x 10^9^ CFU/mL.

### 2.3 DNA isolation and quantitative real-time PCR analysis

Genomic DNA (gDNA) was isolated from 99 clinical tumor tissues using a QIAamp DNA Mini kit (Qiagen, UK) according to the manufacturer’s protocol. Briefly, the samples were suspended with protease K in ATL buffer and incubated at 55°C for 2 hr. Both AL buffer and absolute ethanol were added to the samples before applying the QIAamp spin column. Each sample was centrifuged and washed. DNA was eluted from the column with 50 μL of the supplied AE buffer. The quality and quantity of isolated DNA were determined using a NanoDrop spectrophotometer (ND-1000; Thermo Scientific, USA). All samples were stored at -20°C until used.

FN detection from 99 tumor samples was evaluated by quantitative real-time PCR using the TaqMan assay system, as previously described (Lee et al., 2021). Each reaction was conducted in a final volume of 20 μL reaction containing 1× TaqMan Universal PCR Master Mix (Applied Biosystems, USA), 300 nM of each primer, 200 nM TaqMan probe, and 30 ng of gDNA in a 96-well optical PCR plate. Amplification, detection, and data analysis were performed using the StepOnePlus Real-Time PCR System (Applied Biosystems, USA) as follows: 10 min pre-incubation at 95°C, amplification cycles of 95°C for 15 s, and 60°C for 1 min were repeated 50 times. The cycle threshold (Ct) values for FN were normalized to the amount of gDNA in each reaction, using prostaglandin transporter (PGT) as a human reference gene. The primer and probe sequences were as follows: 16S ribosomal RNA (16s rRNA) gene DNA sequence of FN forward primer, 5′-GGATTTATTGGGCGTAAAGC-3′; FN reverse primer, 5′-GGCATTCCTACAAATATCTACGAA-3′; FN FAM probe, 5′-CTCTACACTTGTAGTTCCG-3′; PGT forward primer, 5′- ATCCCCAAAGCACCTGGTTT-3′; PGT reverse primer, 5′- AGAGGCCAAGATAGTCCTGGTAA-3′; PGT FAM probe, 5′- CCATCCATGTCCTCATCTC-3′.

### 2.4 Total RNA isolation and microarray

Total RNA was prepared from 99 clinical tumor tissues using the RNeasy Mini kit (Qiagen, UK) according to the manufacturer’s protocol. Briefly, clinical samples were lysed with a reagent in a microcentrifuge tube, and 70% ethanol was added to the lysate. After vortexing for 10 s, the lysate was transferred to a RNeasy spin column and centrifuged at 8,000 x *g* for 15 s. The spin columns were then washed with RW1 and RPE buffer. Total RNA was eluted in 50 μL RNase-free water and stored at -80°C until use. The concentrations of total RNA were determined using a NanoDrop spectrophotometer (ND-1000; Thermo Scientific, USA) and RNA integrity number (RIN) values were assessed using Agilent 2100 bioanalyzer (Agilent Technologies, Inc. USA).

For Illumina microarray analysis, 1.5 μg of total RNA was amplified and labeled with biotinylated nucleotides using Illumina Total Prep RNA amplification kit (Ambion, USA) according to previous studies (Kwon et al., 2017). Samples were purified using the RNeasy kit (Qiagen, UK). Hybridization, washing and scanning with Sentrix HumanHT-12 v4 Expression Bead Chip (Illumina, USA) were performed according to the Illumina BeadStation 500× System Manual.

### 2.5 Differentially expressed genes (DEGs) and Gene Ontology (GO) enrichment analysis

Microarray data was extracted, normalized, and analyzed using Illumina BeadStudio provided by the manufacturer. Expression values were then log_10_-transformed. The categorization was carried out depending on whether the gene was upregulated (log_10_ [fold change] ≥ 0.3 and *P*<0.05), downregulated (log_10_ [fold change] ≤ −0.3 and *P*<0.05), or unchanged in FN-positive tumor tissues compared with those of FN-negative tumors.

The identified DEGs were subjected to GO enrichment analysis using the Database for Annotation, Visualization, and Integrated Discovery (DAVID) (http://david.abcc.ncifcrf.gov/). GO terms (“biological processes”) with a *P*-value <0.05 were considered significantly enriched by the DEGs. Only the GO terms with false discovery rate (FDR) <0.10 were considered as significant and ranked by FDR in ascending order.

### 2.6 CMS classification

Consensus molecular subtype (CMS) classification was performed based on the gene level according to a previous study (Guinney et al., 2015). CMS subtypes were classified using the random forest (RF) predictor implemented in the R package “CMSclassifier.” The similarity of the expression profile to the four subtypes (CMS1 to CMS4) was calculated for each case.

### 2.7 Immunohistochemistry (IHC)

Surgical specimens were fixed in 10% buffered formalin for 48 hr and dehydrated with a series of ethanol before being embedded in paraffin. Immunohistochemistry was carried out on a fully automated VENTANA Benchmark XT Stainer (VENTANA Medical Systems; Roche Group, USA), according to a previous study (Kim et al., 2019). The paraffin-embedded tissue blocks were cut into 4 µm sections, mounted on glass slides, deparaffinized, and rehydrated. The sections were incubated with the following primary antibodies: anti-CD-3 (1:200; Cat# IS503, Dako, Denmark) and anti-CD-8 (1:200; Cat# IR623, Dako, Denmark). The staining was developed using the ultraView Universal DAB Detection Kit (Ventana Medical Systems, USA), followed by hematoxylin II counterstain according to the manufacturer’s instructions (Ventana, USA). To measure CD3^+^ or CD8^+^ T lymphocyte density, all stained slides were scanned (magnification ×200) using a VENTANA iScan HT slide scanner (Ventana Medical Systems, USA).

### 2.8 Lymphocyte isolation from tumor tissue and flow cytometry

Lymphocytes were isolated from the tumor tissue by enzymatic dissociation with the human tumor tissue dissociation kit (Miltenyi Biotec, USA) according to the manufacturer’s instructions. The single cell suspension was then filtered through a 70 μm cell strainer, washed with PBS, and centrifuged at 300 x *g* for 5 min. The supernatant was discarded, then stained with the LIVE/DEAD fixable dead cell staining kit (Invitrogen, USA) to remove dead cells. The surface proteins were stained with fluorophore-conjugated antibodies. Intracellular staining was performed with fluorophore-conjugated antibodies and FoxP3 staining buffer kit (Thermo Fisher Scientific, USA). After washing, the cells were analyzed with an LSR II instrument and FlowJo software. The antibodies used for the flow cytometry analysis are listed in **Table 2**. Gating strategies are shown in **Supplementary Figures 3A, B**. First, single cells were discriminated by forward-light scattering signal height (FSC-H) versus forward-light scattering signal area (FSC-A) gating. A lymphocyte gate was gated using a side scattering signal area (SSC-A) versus an FSC-A. We then gated for CD3^+^ T cells on live lymphocytes to exclude dead cells. In the CD3^+^ gating, CD4^+^ or CD8^+^ T cells were further gated on the CD8^+^ versus CD4^+^ plot.

**Table 2:** List of antibodies.

Antibodies	Source	Identifier
Mouse anti-human CD3-BV786, SK7	BD Biosciences	Cat# 563800
Mouse anti-human CD3-V500, UCHT1	BD Biosciences	Cat# 561416
Mouse anti-human CD45RA-FITC (HI100)	Thermo Fisher Scientific	Cat# 11-0458-42
Mouse anti-human CD4-APC-H7, RPA-T4	BD Biosciences	Cat# 560158
Mouse anti-human CD4-APC-R700, RPA-T4	BD Biosciences	Cat# 564975
Mouse anti-human CD8, C8/144B	Dako	Cat# IR623
Mouse anti-human CD8-APC-R700, RPA-T8	BD Biosciences	Cat# 565165
Mouse anti-human FoxP3-PE-Cyanine7, PCH101	Thermo Fisher Scientific	Cat# 25-4776-42
Mouse anti-human PD-1 (CD279)-Brilliant Violet 421^TM^, EH12.2H7	BioLegend	Cat# 329920
Mouse anti-human TIGIT-PerCP-eFluor710, MBSA43	Thermo Fisher Scientific	Cat# 46-9500-42
Mouse anti-human TIM-3 (CD366)-BV711, 7D3	BD Biosciences	Cat# 565566
Rabbit anti-human CD3, polyclonal	Dako	Cat# IS503

### 2.9 T cell stimulation with or without the presence of FN

T cells from FN-negative tumors were stimulated for 48 hr in RPMI1640 (2 mM L-glutamine) containing 10% fetal bovine serum (FBS) in the 24-well plates coated with anti-CD3 antibody. During stimulation, cells were infected with FN at a multiplicity of infection (MOI) of 100 for 48 hr at 37°C in a humidified air atmosphere containing 5% CO_2_. After incubation, cells were harvested and stained with anti-CD3, anti-CD4, anti-CD8, anti-FOXP3, anti-PD-1, anti-TIM-3, and anti-TIGIT antibodies. The stained cells were analyzed by flow cytometry.

### 2.10 Statistical analysis

Data analyses were performed using GraphPad Prism 8.0 and R statistical software. Paired or unpaired *t*-tests were used to compare continuous variables. The chi-squared test was used to compare qualitative variables. Pearson or Spearman correlation analyses were used to evaluate correlations between parameters. The one-tailed Fisher’s exact test was used to assess the significance of overlapping genes between different groups and the log-rank test to compare survival outcomes. Significance was set at *P*<0.05.

## 3 Results

### 3.1 Distribution of CMS subtypes and outcomes according to FN infection

**Figure 1:**
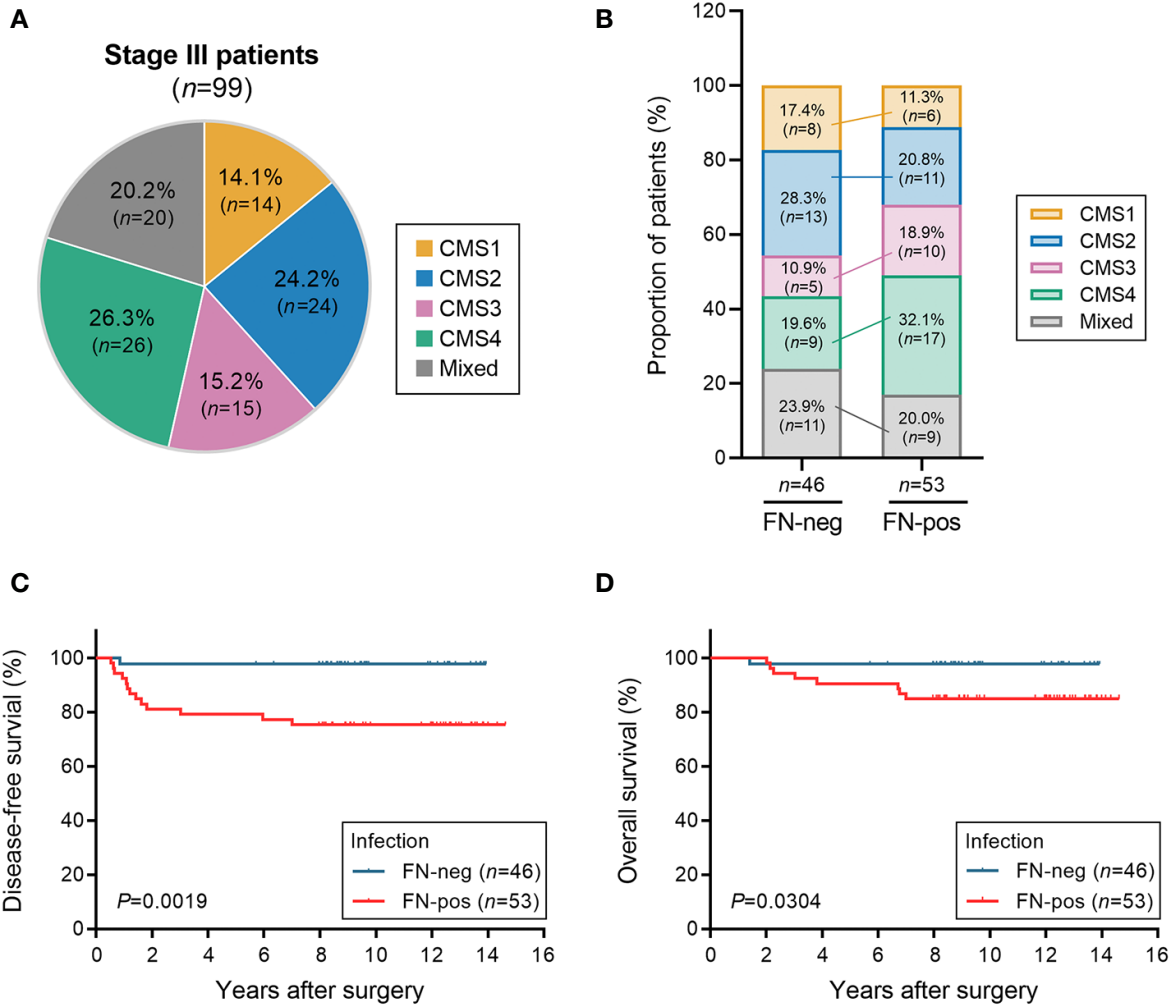
Association of FN infection, consensus molecular subtype (CMS) groups, and survival. **(A)** The distribution of the consensus molecular subtypes (CMS) groups in patients with stage III CRC. **(B)** Proportion of the CMS group in patients with CRC according to FN infection. **(C, D)** Disease-free survival **(C)** and overall survival **(D)** according to FN infection. Statistical tests: *P < 0.05, **P < 0.01.

We analyzed tumor tissues of 99 patients with stage III colorectal carcinoma cases (**Table 1**). Our cohort was predominantly comprised of left-sided colon cancers (64.6%) and microsatellite stable cancers (91.9%). *KRAS* mutation and *BRAF* mutations were found in 40 (40.4%) patients and 1 (1.0%) patient, respectively. To investigate the classification of CMS subtypes in tumor tissues, we analyzed the mRNA expression patterns using microarray. Patients were classified as CMS1 (14.1%), CMS2 (24.2%), CMS3 (15.2%), CMS4 (26.3%), and mixed (20.2%) (**Figure 1A**). Real-time quantitative PCR was used to detect FN infection in tumor tissues. Of the 99 patients with CRC, 53 (53.5%) were positive for FN infection and 46 (46.5%) were negative. The proportion of CMS subtypes in 99 patients was examined according to the FN infection (**Figure 1B**). Interestingly, mesenchymal subtype (CMS4) was the most prevalent in FN-positive patients (32.1%). Next, we assessed the relationship between FN infection and patient outcomes in disease-free survival (DFS) and overall survival (OS) using the Kaplan–Meier method (**Figures 1C, D**). Both DFS and OS were lower in patients with FN-positive compared to those FN-negative (*P*=0.0019 for DFS and *P*=0.0304 for OS). CMS groups were not associated with survival outcomes in our cohort (**Supplementary Figures S1A, B**).

### 3.2 Transcriptional changes in tumor tissues according to FN infection

**Figure 2:**
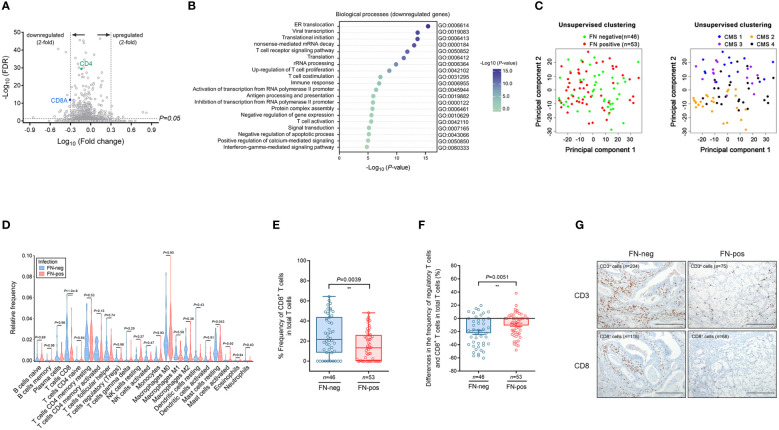
Expression profile and immune cell infiltration of colorectal cancer according to FN infection. **(A)** Volcano plot of differentially expressed genes (DEGs) according to FN infection. **(B)** Gene ontology analysis of the top 20 biologic processes negatively enriched in FN-positive tumor tissues. **(C)** Unsupervised principal component analysis (PCA) according to FN infection or CMS groups among samples. **(D)** Immune cell composition of tumor tissues according to FN infection based on deconvolution method. **(E)** Relative frequency of CD8^+^ T cells in total T cells of tumor tissues according to FN infection based on deconvolution method. **(F)** Differences in the frequency of regulatory T cells and CD8^+^ T cells in total T cells of tumor tissues according to FN infection based on the deconvolution method. **(G)** Immunohistochemistry for T cell infiltration of tumor tissues according to FN infection. Scale bars, 100 μm. Error bars indicate SEM. Statistical tests: *P < 0.05, **P < 0.01, ****P < 0.0001.

We performed microarray analysis of tumor tissues and a total of 563 differentially expressed genes (DEGs) were identified by comparing FN-positive tumor tissues and those of FN-negative tumors (corrected *P*-value <0.05). We found 436 down-regulated genes and T-cell transcript genes, including CD3E, CD4 and CD8A (**Figure 2A**). Among the 127 upregulated DEGs, we identified *FCGR2B*, *FCGR3A*, *ADAR*, *WASL*, *SDC4*, *ADCY9*, *ZBTB7B*, *PAG1*, *CEBPB*, *VIPR1*, and *POMC* involved in the negative regulation of lymphocyte proliferation and activation. We hypothesized that FN infection would be related to the depletion of T cells in the tumor microenvironment.

To determine this possibility, we performed a GO term analysis of biological processes using DEGs identified by FN infection. Downregulated DEGs were involved in immune responses, including T cell receptor signaling pathway, T cell co-stimulation, T cell activation, and interferon-gamma-mediated signaling pathway (**Figure 2B**). In contrast, upregulated DEGs were enriched in the pathways related to transcription and differentiation (**Supplementary Figure S2**).

Unsupervised principal component analysis (PCA) was used to cluster the samples in terms of gene expression profiling (**Figure 2C**). PCA plots show that the colon tumor tissues are not distinct in terms of FN-positive or FN-negative classes whereas the samples are largely segregated in accordance to the CMS classification.

Next, we examined 21 immune cell types in 99 tumor tissues by using the CIBERSORT analytical deconvolution tool (**Figure 2D**). Interestingly, the proportions of CD8 T cells in FN-positive tumor tissues were lower than those in FN-negative tumors (*P*<0.001). The T follicular helper cells, M1 macrophages, activated dendritic cells, and neutrophils exhibited a decreasing tendency according to FN infection, but there was no significance. On the contrary, the proportions of mast cells resting (*P*=0.043) were higher than those in FN-negative tumors.

To determine whether CD8^+^ T cells in tumors exhibit any exhausted phenotype in response to FN infection, we examined the relative expression of CD8^+^ T cells among total tumor T cells (**Figure 2E**). The proportion of CD8^+^ T cells among total T cells was lower in FN-positive tumors compared with FN-negative samples (*P*=0.0039). We also assessed differences in the frequency of regulatory T cells and CD8^+^ T cells in total T cells (**Figure 2F**). The differences in the frequency of regulatory T cells and CD8^+^ T cells were diminished by FN infection (*P*=0.0051).

Then, immunohistochemical staining for CD3 and CD8 was performed to assess T-cell infiltration in tumor tissue. We observed that the CD3^+^ and CD8^+^ cells were reduced in FN-positive tumor tissues compared to FN-negative tumors (**Figure 2G**).

Our findings suggest that poorer prognosis in CRC patients is probably derived from T cell-mediated immune responses by FN infection.

### 3.3 Association of FN infection with T cell composition in tumor tissues

**Figure 3:**
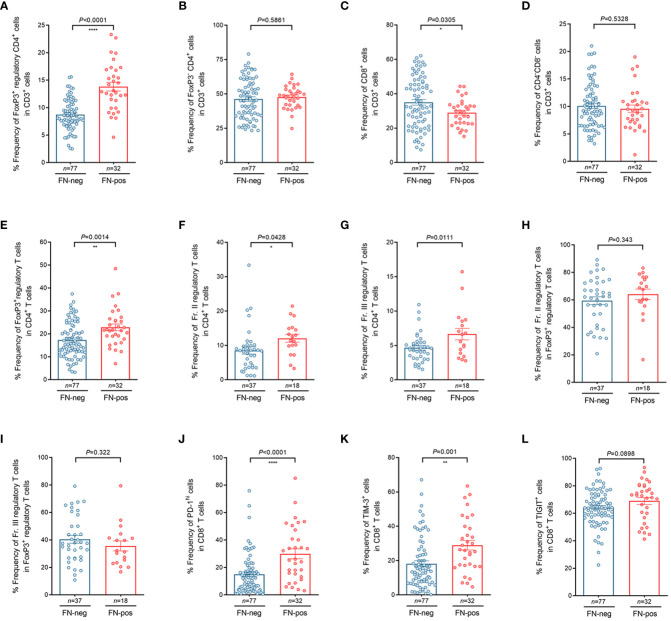
Characteristics of tumor-infiltrating regulatory T cells and CD8^+^ T cells according to FN infection. **(A–D)** Relative frequency of FoxP3^+^CD4^+^ regulatory T cells **(A)**, FoxP3^-^ CD4^+^ T cells **(B)**, CD8^+^ T cells **(C)**, and CD4^-^CD8^-^ T cells **(D)** in tumor-infiltrating CD3^+^ T cells. **(E–I)** Relative frequency of FoxP3^+^ regulatory T cells in tumor-infiltrating CD4^+^ T cells **(E)**, FoxP3^hi^CD45RA^lo^ regulatory T cells (Fr. II) in tumor-infiltrating CD4^+^ T cells **(F)**, FoxP3^lo^CD45RA^lo^ regulatory T cells (Fr. III) in tumor-infiltrating CD4^+^ T cells **(G)**, FoxP3^hi^CD45RA^lo^ regulatory T cells (Fr. II) in tumor-infiltrating FoxP3^+^ regulatory T cells **(H)**, FoxP3^lo^CD45RA^lo^ regulatory T cells (Fr.III) in tumor-infiltrating FoxP3^+^ regulatory T cells **(I)**. **(J–L)** Relative frequency of PD-1^hi^ **(J)**, TIM-3^+^ **(K)**, and TIGIT^+^ **(L)** cells in tumor-infiltrating CD8^+^ T cells. Error bars indicate SEM. Statistical tests: *P < 0.05, **P < 0.01, ****P < 0.0001.

To investigate whether FN infection contributes to T cell composition, we performed flow cytometry analysis for tumor-infiltrating lymphocytes (TILs) derived from tumor tissues in patients with mismatch repair-proficient CRC. In the analysis of CD3^+^ T cells, we found that the relative frequency of FoxP3^+^CD4^+^ regulatory T cells among CD3^+^ T cells were higher in FN-positive tumors, whereas the relative frequency of CD8^+^ T cells among CD3^+^ T cells were lower in this group (**Figures 3A–D**). We also found that the relative frequency of regulatory T cells (FoxP3^+^CD4^+^ T cells) was higher in FN-positive tumor tissues than in those FN-negative tumors in the analysis of CD4^+^ T cells (**Figure 3E**, *P*=0.0014). Next, we examined the frequencies of FoxP3^+^ T cell subpopulations (Fr. II, FoxP3^hi^CD45RA^-^ and Fr. III, FoxP3^low^CD45RA^-^) in tumor tissue according to FN infection. Among the CD4^+^T cell population of TILs, the frequencies of both Fr. II (**Figure 3F**, *P*=0.0428) and Fr. III (**Figure 3G**, *P*=0.0111) regulatory T cells were significantly increased in FN-positive tumor tissues compared to those FN-negative tumors, respectively. The association of FN infection with Fr. II and Fr. III regulatory T cells in FoxP3^+^ regulatory T cells in tumor tissues was not statistically significant (**Figures 3H, I**; *P*=0.343 and *P*=0.322, respectively).

We further tested whether FN infection affects immune checkpoint inhibitory receptors including PD-1, TIM-3, and TIGIT in CD8^+^ TILs. Interestingly, we observed that the frequencies of PD-1^hi^ CD8^+^ T cells (**Figure 3J**, *P*<0.0001) and TIM-3^+^ CD8^+^ T cells (**Figure 3K**, *P*=0.001) were higher in FN-positive tissues. **Figure 3L** shows that the frequencies of TIGIT^+^ CD8^+^ T cells tended to increase in FN-positive tumor tissues, but there was no significant difference between FN-positive and FN-negative tumor tissues (**Figure 3L**, *P*=0.0898). To investigate whether the FN burden is associated with immune phenotypes, FN-positive samples (*n*=32) were categorized into low- and high-burden FN (*n*=16 for each group). Our results showed that patients with a high burden of FN exhibited a higher frequency of FoxP3^+^ regulatory T cells among CD4^+^ T cells, compared to negative controls (**Supplementary Figure 4A**, *P*=0.0013). We also observed that FN burden affects immune checkpoint inhibitory receptors including PD-1, TIM-3, and TIGIT in CD8^+^ TILs. The frequencies of PD-1^hi^ CD8^+^ T cells (**Supplementary Figure 4B**, *P*<0.0001), TIM-3^+^ CD8^+^ T cells (**Supplementary Figure 4C**, *P*=0.0005), and TIGIT^+^ CD8^+^ T cells (**Supplementary Figure 4D**, *P*=0.0203) were relatively increased in high-burden FN samples compared to FN-negative samples. There was no significant difference between low burden FN samples and negative controls.

Our results suggest that FN contributes to the generation of an immunosuppressive tumor microenvironment (TME) by inducing a disproportionate T cell composition in colorectal carcinoma tissues.

### 3.4 The direct interactions between FN infection and immune checkpoint inhibitory receptors in T cells

**Figure 4:**
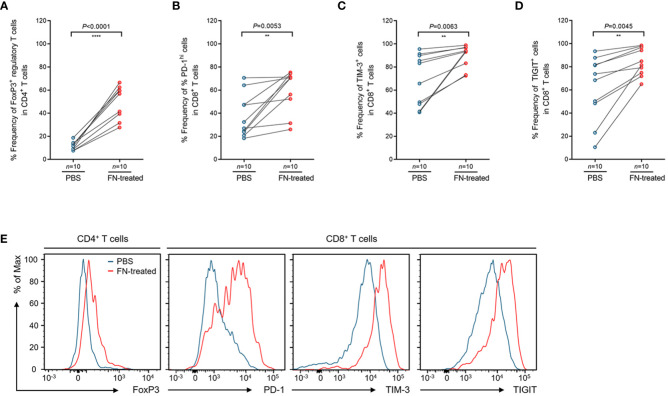
Phenotypic changes of tumor-infiltrating regulatory T cells and CD8^+^ T cells upon FN treatment. **(A)** Relative frequency of FoxP3^+^ regulatory T cells in tumor-infiltrating CD4^+^ T cells upon FN infection. **(B–D)** Relative frequency of PD-1^hi^ **(B)**, TIM-3^+^ **(C)**, and TIGIT^+^ **(D)** cells in tumor-infiltrating CD8^+^ T cells upon FN treatment. **(E)** A representative histogram showing the expression of FoxP3, PD-1, TIM-3, and TIGIT for tumor-infiltrating T cells. Error bars indicate SEM. Statistical tests: **P < 0.01, ****P < 0.0001.

To assess whether FN infection directly induces T cell-mediated immune responses in TILs, *ex vivo* stimulation experiment was performed. We isolated TILs from FN-negative tumor tissues. Single cell suspension of tumor tissues was prepared and then stimulated by anti-CD3 antibody. During stimulation, single cell suspension was infected with FN at a MOI of 100:1 (bacteria: cells) for 48 hr. The control group was treated with PBS instead of FN. Similar results to those shown in **Figure 3** were observed from the T cell stimulation system. In FN-treated group, the frequencies of FOXP3^+^CD4^+^ T cells were significantly increased compared to control group (**Figure 4A**, *P*<0.0001). We next examined the expression of immune checkpoint inhibitory receptors (PD-1, TIM-3, and TIGIT) in CD8^+^ T cells according to FN infection. Interestingly, PD-1 (*P*=0.0053), TIM-3 (*P*=0.0063), and TIGIT (*P*=0.0045) were highly expressed in CD8^+^ T cells after treatment with FN (**Figures 4B–D**). Representative histograms presenting these data are presented in **Figure 4E**, showing increased expression of CD4^+^ FoxP3^+^ T cells, CD8^+^ PD-1^+^, CD8^+^ TIM-3^+^, and CD8^+^ TIGIT^+^ T cells in FN-positive tumors. These findings provide evidence that FN infection is capable of inducing an immunosuppressive tumor microenvironment in patients with colorectal cancer.

## 4 Discussion

Most stage III patients with CRC are usually treated with routine treatment to reduce the risk of disease progression, even though 50% of the stage III patients are cured by surgery alone, 20% with the treatment of adjuvant chemotherapy, and 30% of recurrence (Auclin et al., 2017). A better understanding of tumor biology and prognostic factors in patients with CRC can provide potential strategies for therapeutic intervention. Interactions between the gut microbiota and the immune system affect cancer progression and treatment of patients (Belkaid and Hand, 2014; Sears and Garrett, 2014; Zheng et al., 2020).

Genus of *Fusobacterium*, *Bacteroides*, *Parvimonas*, and *Prevotella* are known to be pathogenic bacteria in colorectal cancer (York, 2017; Omar Al-Hassi et al., 2018). *Fusobacterium nucleatum* (FN) is frequently found in tumors and related to the prognosis of colorectal cancer (Castellarin et al., 2012; Kostic et al., 2012). High levels of FN have been identified to promote tumor progression by changing the mucosal microbiota and the transcriptional activity of tumor-related metabolic pathways (Wu et al., 2022). Two previous studies have shown that FN promotes chemoresistance by regulating apoptosis and autophagy pathways (Yu et al., 2017; Zhang et al., 2019). Recently, Viljoen et al. showed that levels of FN and enterotoxigenic *Bacteroides fragilis* (ETBF) were significantly higher in advanced CRC (stages III and IV) and that in particular there was a positive correlation between regional lymph node metastases and high levels of *Fusobacterium* colonization (Viljoen et al., 2015). This is similar with a previous study (Gethings-Behncke et al., 2020), which reports that the high presence of FN in tumor tissue was associated with poorer overall survival (OS) in patients with CRC. In the present study, we found that FN infection was associated with poorer outcomes in stage III CRC patients.

The gene expression-based consensus molecular subtype (CMS) classification is able to predict patient survival for advanced CRC (Guinney et al., 2015). In particular, CMS4 (Mesenchymal) group was linked to a higher risk of disease recurrence in adjuvant-treated patients (Auclin et al., 2017). Previous studies have demonstrated that FN and other bacteria are frequently found in CMS1 patients compared with CMS2–4 patients (Salvucci et al., 2022). They have shown that when CRC patients are classified as mesenchymal (CMS4) versus non-mesenchymal (neither CMS4), the risk of poor prognosis in patients with mesenchymal tumors and high-abundance FN is approximately two-fold. In our study, the CMS4 group was prevalent in FN-positive tissues in patients with stage III CRC, but there was no significant difference in survival between the CMS groups according to FN infection. Our previous study has shown that high abundance of FN has significantly poorer prognosis in terms of progression-free survival 1 in right-sided metastatic colon cancers (Lee et al., 2021). In a recent study, CMS4 tumors are characterized by high expression of immune response genes and a marked immune infiltration (Becht et al., 2016). We investigated the association between immune response and FN infection in tumor tissues by gene expression analysis. FN infection was found to be related to T cell responses, including T cell receptor signaling, T cell costimulation, T cell proliferation and T cell activation.

Our results showed that the relative frequency of CD8 T cells was significantly different between FN-negative and FN-positive tumors. Low densities of CD3^+^ T cells are found in FN-infected tumor tissues (Mima et al., 2015). Our results have shown that the densities of CD3^+^ T cells was significantly decreased in FN-positive tumor tissues compared with those FN-negative tumors. In a study by Jorge Luis Galeano Niño et al., *Fusobacteria* were identified as the most dominant genera in the CRC tumor tissues by quantifying UMI metric of specific organisms *via* a spatial transcriptomics platform (Galeano Nino et al., 2022). They showed that bacteria-positive areas of tumor tissue had lower densities of CD4^+^ and CD8^+^ T cells, as well as increased CD11b^+^ and CD66b^+^ myeloid cells, compared with bacteria-negative areas. This indicates that the *Fusobacteria* are highly associated with the immune modulation in the TME. Virulence factors of pathogens interact directly with the host immune system. For example, Fap2 has been identified as a virulence factor of FN and interacts with the Gal/GalNAc sugar residues of tumor cells (Kaplan et al., 2010; Casasanta et al., 2020). A study shows that NK cell activity is inhibited by the Fap2 protein of FN (Gur et al., 2015). Our findings demonstrate that host–microbiota interactions contribute to T cell differentiation and function.

T cell-mediated adaptive immunity is considered to play a major role in antitumor immunity (Galon et al., 2014). Regulatory T cells (Tregs) are a specialized subpopulation of T cells that express the FoxP3 transcription factor and are involved in antitumor immunity (Miyao et al., 2012; Bending et al., 2018). Patients with CRC with FoxP3^hi^ regulatory T cells had a significantly poor prognosis than patients with FoxP3^lo^ regulatory T cells (Saito et al., 2016). Tumors with higher levels of CD3^+^, CD8^+^ and CD45RO^+^ T lymphocytes are associated with better patient survival (Nosho et al., 2010). In the flow cytometry analysis, we found that the relative frequency of FoxP3^hi^ regulatory T cells was increased in FN-positive tumor tissues compared to those of FN-negative tumors. In contrast, lower frequencies of CD8^+^ T cells among CD3^+^ T cells were observed in FN-positive tumor tissues. Our results have shown that FN induces downregulation of the T cell response in CRC.

Growing evidence has shown that tumor progression and immune evasion occur by the T cell-mediated immune response in the TME (Ostrand-Rosenberg et al., 2014; Masugi et al., 2017). An immune score of colorectal cancer could be considered as a predictor of response to chemotherapy in patients with stage II and III (Galon et al., 2014). The efficacy of immunotherapy is closely related to the expression of immune checkpoints including PD-1, TIM-3, and TIGIT (Qin et al., 2019). In the TME, PD-1 and Tim-3 promote cancer immune evasion through T cell depletion (Jiang and Li, 2015). TIGIT directly interacts with Fap2, the outer membrane protein of FN, leading to inhibition of the cytotoxic activity of natural killer (NK) cells against cancer cells (Gur et al., 2015). In accordance with this evidence, we observed that PD-1, TIM-3, and TIGIT in TILs were increased in FN-positive tumor tissues compared to those FN-negative tumors. To identify the direct interaction between T cell response and FN infection, we performed an *ex vivo* T cell stimulation assay. We found that the presence of FN in TILs affected directly the T cell responses and immune checkpoint inhibitory receptors. A recent study has shown that the presence of FN enhances the efficacy of PD-L1 blockade in CRC by activating STING signaling (Gao et al., 2021). Hence, FN infection could alter the tumor microenvironment by modulating immune cells in a way that allows rapid evolution and escape of the cancer cells. Further study is required to provide evidence of the direct influence of FN on T cell immunity and immune checkpoint inhibitory receptors.

Although this study showed a correlation between the FN infection and an immunomodulatory effect in CRC, the potential role of other gut bacteria in immunomodulation was suggested. Immune cell analysis showed that *Bifidobacterium* strains enhanced antitumor immunity by increasing CD8^+^ T cell proliferation and IFN-gamma production (Sivan et al., 2015). *In vitro* and *in vivo* studies have demonstrated that the presence of *Lacticaseibacillus paracasei sh2020* improved the poorly infiltrated TME by increasing CD8^+^ T cell recruitment and T cell infiltration (Zhang et al., 2022). This suggests that microbial dysbiosis can induce T cell exhaustion during the development of tumors in the colon. Therefore, further studies are elusive to understand how pathogenic gut microbiota changes affect T cell-associated immune responses in the development of CRC.

Treatment with antibiotics reduces the growth of FN-positive tumors *in vivo* (Bullman et al., 2017). However, FN may persist in endosomes and lysosomes, as frequent antibiotic use may induce FN internalization into tumor, immune, and endothelial cells. The persistence of FN can reduce T-cell activity in tumors. Thus, antibiotic therapy may be limited in treating FN-positive CRC tumors. The development of strategies for the eradication of FN or regulation of T cells could help improve clinical outcomes in patients with CRC.

In conclusion, we found that FN infection was associated with poor prognosis in CRC patients by modulating T cell-mediated immune responses. It will need to be further validated in a large cohort and to elucidate the details of the immunosuppressive mechanism of FN in cancer progression.

## Data availability statement

The original contributions presented in the study are included in the article/**Supplementary Material**. Further inquiries can be directed to the corresponding author.

## Ethics statement

The studies involving human participants were reviewed and approved by the institutional review board of Severance Hospital (IRB no: 4-2018-0291). The patients/participants provided their written informed consent to participate in this study.

## Author contributions

The authors HSK, CGK, WKK contributed to conceptualization and design of this study, data curation, and development of methodology. KK and JY performed the formal statistical analysis and software implementation. HSK, CGK, KK wrote the original draft manuscript. BSM provided the patient tissue samples. SP, SJS, HL, KL, and HK reviewed and edited the manuscript. ES, TK, and JBA supervised the study. All authors contributed to the article and approved the submitted version.

## Supplementary material

Supplementary Figure 1 Association of consensus molecular subtype groups and survival. **(A, B)** Disease-free survival **(A)** and overall survival **(B)** according to consensus molecular subtype groups.

Supplementary Figure 2 Gene Ontology (GO) analysis of top 20 biologic processes positively enriched in FN-infected CRC tumor tissue.

Supplementary Figure 3 Examples of gating strategies used to identify CD3^+^ T cells, CD4 ^+^ T cells, and CD8^+^ T cells in human peripheral blood and tumor tissues. **(A, B)** Human peripheral blood **(A)** and tumor tissues **(B)**.

Supplementary Figure 4 Characteristics of tumor-infiltrating regulatory T cells and CD8^+^ T cells according to FN burden. **(A–D)** Relative frequency of FoxP3^+^ regulatory T cells in tumor-infiltrating CD4^+^ T cells **(A)** upon FN burden. Relative frequency of PD-1^hi^ **(B)**, TIM-3^+^ **(C)**, and TIGIT^+^ **(D)** cells in tumor-infiltrating CD8^+^ T cells upon FN burden. Statistical tests: **P* < 0.05, ***P* < 0.01, *****P* < 0.001.
