# Supplementary material for: Fusobacterium nucleatum induces a tumor microenvironment with diminished adaptive immunity against colorectal cancers
Source: Front Cell Infect Microbiol. 2023 Mar 7;13:1101291. doi: 10.3389/fcimb.2023.1101291 (PMC10028079; doi:10.3389/fcimb.2023.1101291)

Supplementary Figure 1

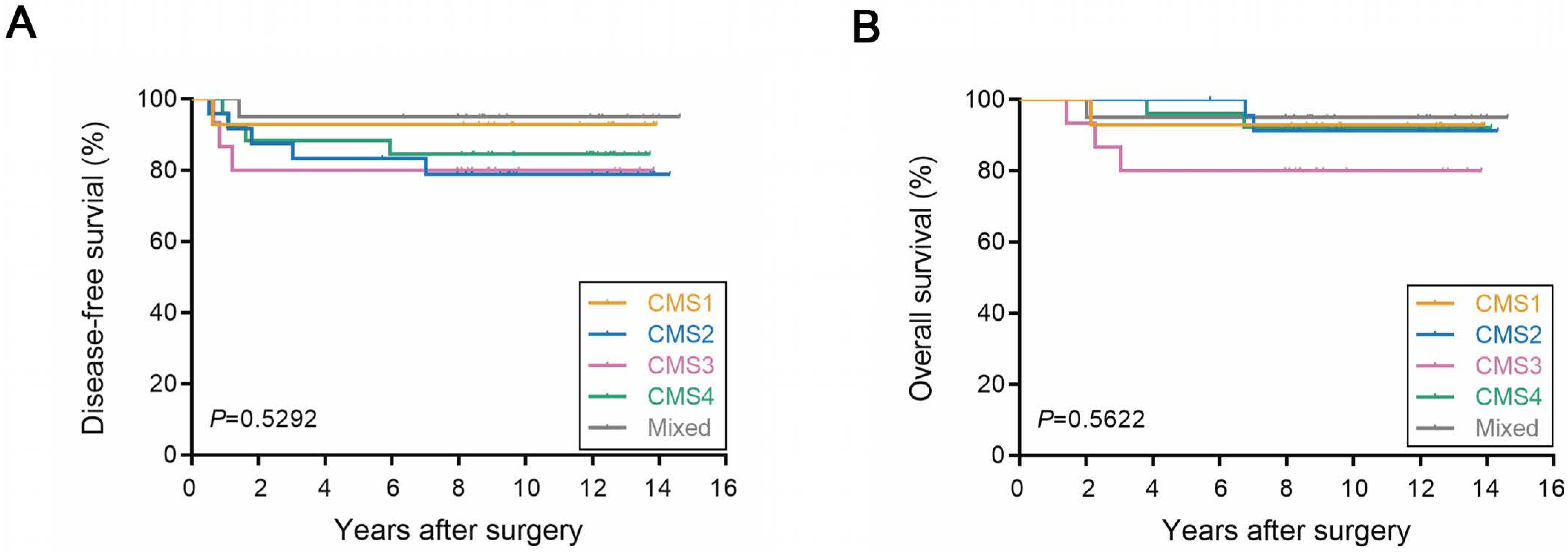

Supplementary Figure 2

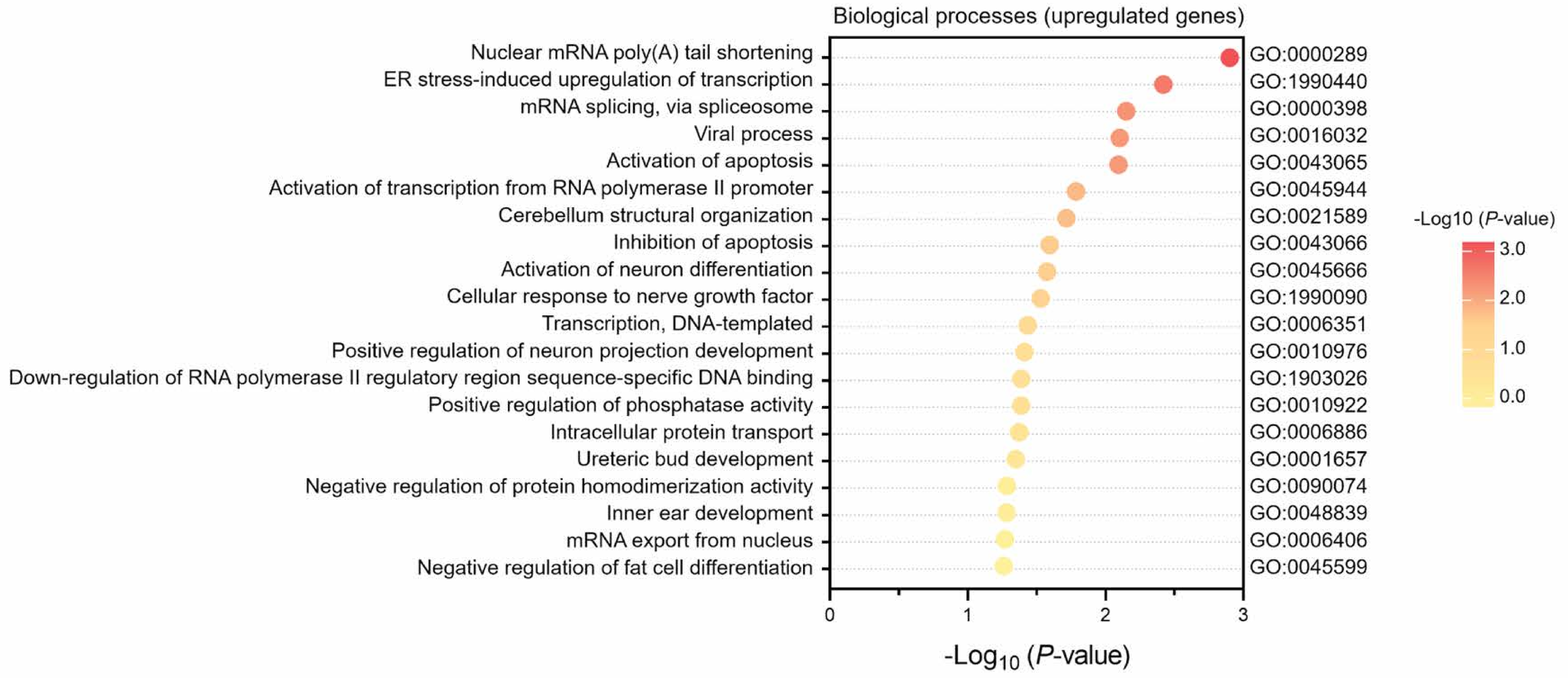

Supplementary Figure 3

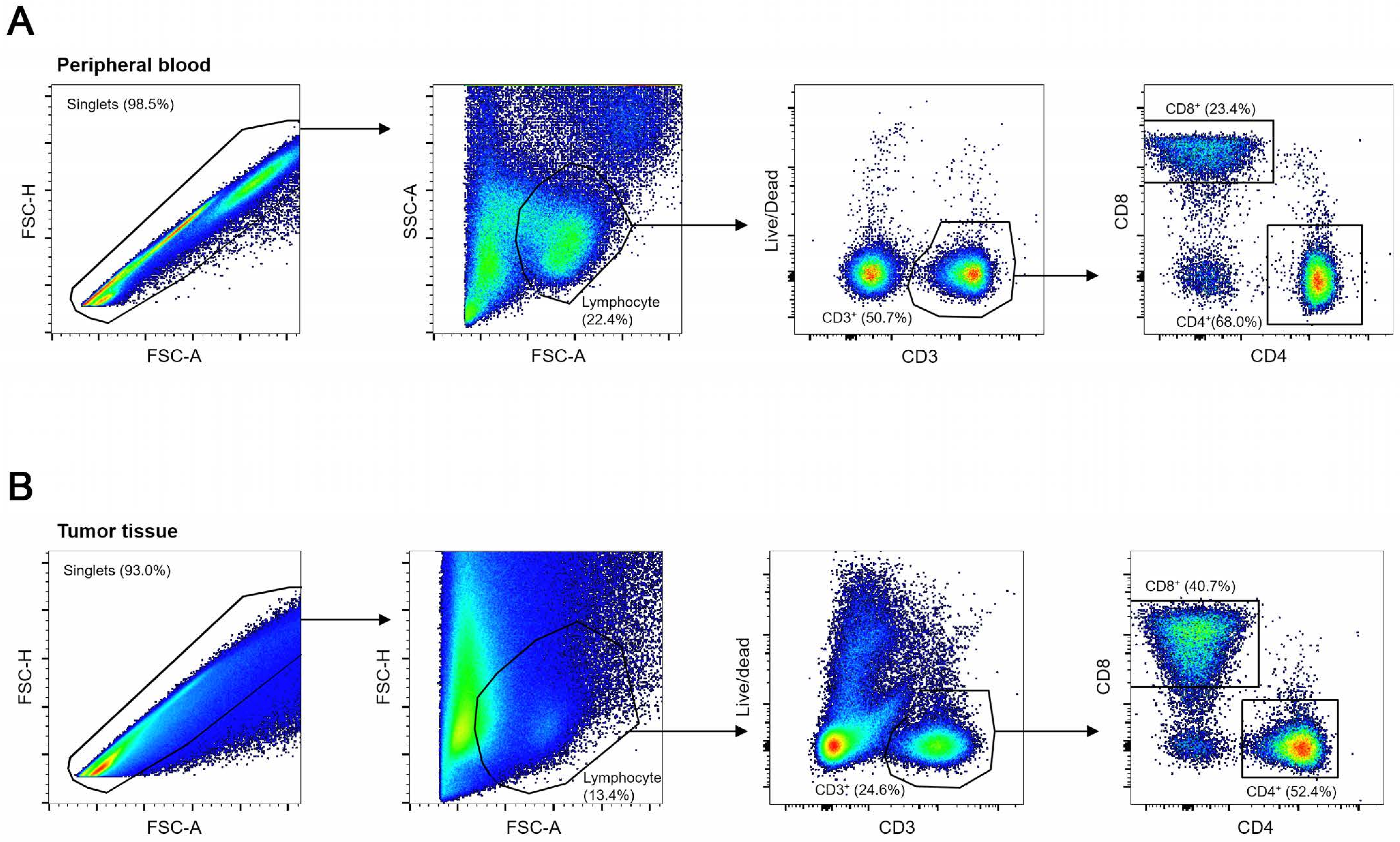

Supplementary Figure 4

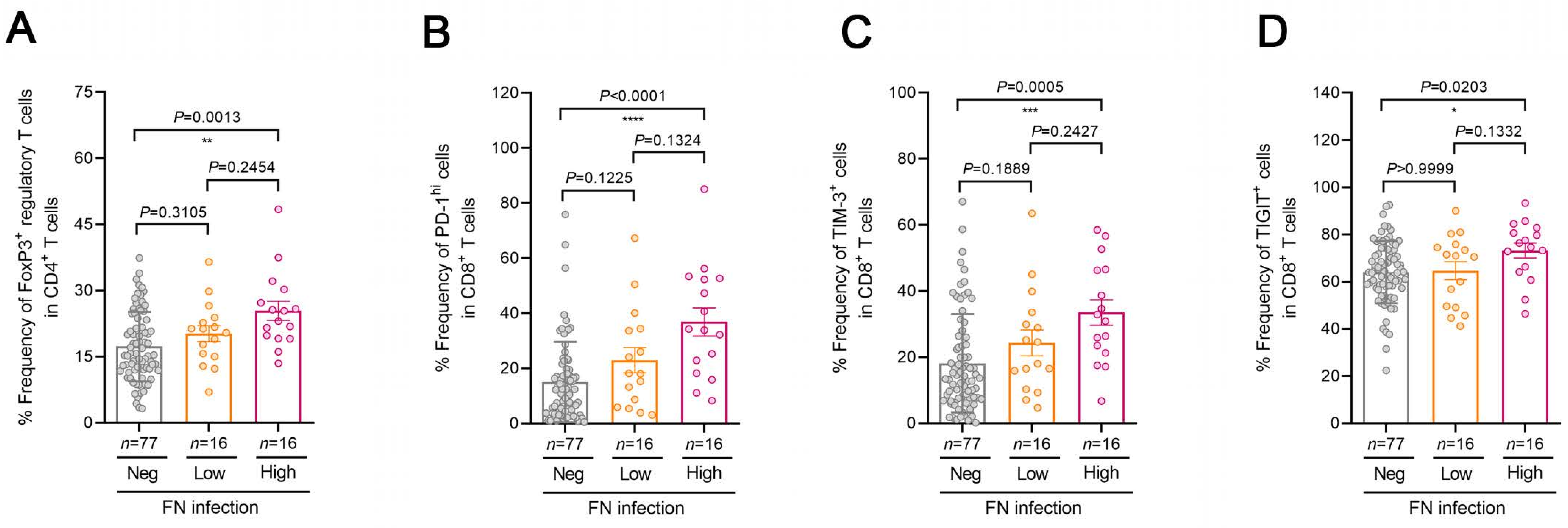

Supplement: Supplementary Figure 1 — Association of consensus molecular subtype groups and survival. (A, B) Disease-free survival (A) and overall survival (B) according to consensus molecular subtype groups. [file Image_1.pdf]
